# Larvicidal effect of Endod (*Phytolacca dodecandra*) seed products against *Anopheles arabiensis* (Diptera: Culicidae) in Ethiopia

**DOI:** 10.1186/s13104-017-2792-5

**Published:** 2017-09-06

**Authors:** Ayalew Jejaw Zeleke, Bezuayehu Alemayehu Shimo, Delenasaw Yewhalaw Gebre

**Affiliations:** 1 Department of Parasitology, School of Biomedical and Laboratory Sciences, College of Medicine and Health Sciences, University of Gondar, Gondar, Ethiopia; 2 Department of Public Health, College of Health Sciences, Mizan Tepi University, Mizan Teferi, Ethiopia; 3 Department of Biology, College of Natural Sciences, Jimma University, Jimma, Ethiopia

**Keywords:** Endod, *Phytolacca dodecandra*, Larvicidal, Lethal concentration

## Abstract

**Objective:**

The purpose of the present study was to determine the larvicidal effect of ‘Endod’ (*Phytolacca dodecandra*) seed products on *Anopheles arabiensis*, in Ethiopia.

**Results:**

Experimental study was conducted using a total of 2400 third instars larvae of *A. arabiensis*. The seed products *P. dodecandra* showed larvicidal activity against 3rd-stage larvae of both the laboratory and field population of *A. arabiensis*. The LC_99_ values for *P. dodecandra’s* seed powder and its extract form against the laboratory reared larvae were 121.07, and 616.46 mg/l, respectively. The LC_50_ and LC_95_ values were also determined.

## Introduction

Globally, an estimated 3.4 billion people were at risk of malaria with 627,000 malaria deaths in 2012. Out of the reported deaths, 90% occurred in SSA and the majority of these were children within the age of <5 years old [1, 2].

Malaria control strategies set out in Africa include on time treatment of clinical attack of malaria with an efficient anti-malarial drug along with proper usage of insecticides for vector control methods. However, its control achievement seems difficult due to the emergence of insecticide and antimalarial drug resistances [2, 3]. Furthermore, majority of the chemical insect killers are hurtful to man and animals [4].

In Ethiopia, population of *Anopheles arabiensis*, which is the major malaria transmitter vector, developed resistance to the conventional insecticides (dichlorodiphenyltrichloroethane, permethrin, deltamethrin and malathion) [5, 6]. The emergence and widespread of insecticide resistance malaria vectors and the dreadful environmental impact of conventional insecticides emphasized the call for the development of alternative malaria control approaches [7].

*Phytolacca dodecandra*, which is commonly called as ‘Endod’ in Ethiopia, is one of among the most important plants in the country. It is also one of the most hopeful plant pesticides because of its high toxicity to the snails, and low toxicity to mammals [8]. However, as per of our knowledge, its toxicity was not investigated for its larvicidal effect against *A. arabiensis*, the major malaria vector in Ethiopia. Therefore, this study aimed to evaluate the larvicidal effect of its seed powder and crude extract form on this malaria vector.

## Main text

### Methods

#### Study setting

An experimental study was conducted in Jimma Univesrsity tropical and infectious diseases research centre from November to February 2016, Jimma, Ethiopia.

#### Sample collection and seed product preparation

Sample from ‘Endod’ (*P. dodecandra*) plant was collected in Mizan Aman town (220 km far away from Jimma). The plant species identification was confirmed and voucher of the specimen has been deposited at the National Herbarium in Addis Ababa University, Ethiopia. Then, berries were collected, washed and dried in the shade place. Finally, they were crushed and powdered using a mortar and pestle. The powder was stored in a dry shaded plastic container for several days prior to use in the laboratory trials [9–11]. Weights of the powder were measured using digital balance in milligram (mg). Accordingly, 0.5, 1, 2, 3, 4 and 5 mg powder were prepared and each were added to 100 ml distill water to form 5, 10, 20, 30, 40, and 50 mg/l concentrations. In order to prepare test concentrations of the extract form, stock solution was obtained by adding 200 mg of the powder into 20 ml distill water. It was kept in a screw-cap vial for 24 h and shacked vigorously to dissolve the powder in the solvent and sieved using Whatman Paper. Then, the filtered liquid solutions were serially diluted and 6 test concentrations were obtained by adding 0.05, 0.10, 0.20, 0.30, 0.40 and 0.50 ml from stock solutions to containers of having 100 ml distilled water as it is described previously [9].

#### Larvae collection/rearing and bioassay

The eggs of *A. arabiensis* mosquito were kept and developed to larvae under standard conditions (25 °C ± 2 °C, 80% ± 4% RH) [12]. Besides, weekly surveys were conducted from stagnant water, and ponds. Larval collection was done using the standard dipping method with a 350 ml mosquito scoop. Morphological identification was done using the previously described mosquito identification key [13]. Then, 3rd-stage larvae were collected in plastic whirl-packs by trained workers from mosquito breeding sites and were transported to the laboratory. They were placed into plastic trays to be reared and feed crushed yeast and wheat flour. The trays had a capacity of holding 3 l (liter) distilled water at 5 cm depth. Finally, 2400 larvae were reared in four trays (600 larvae per tray) by following standard methods [9].

After the larvae were being collected/reared, bioassay was performed. It was performed by using six treatment levels for each of the studied seed products. During the experiment, batches of 25 larvae were exposed to each test concentrations. Identical larval containers were used throughout the experiment and were filled by 100 ml of distill water. The depth of the water in the container was 5 cm. The larvae were transferred from the plastic trays to the container by using dropper and offered a small amount of crushed yeast and wheat flour. The powder and crude extract forms of the seed products were applied onto the water surface of the larval containers. Each test was monitored from the time of application to trial period (24 h) and the numbers of dead larvae were recorded. Dead parts were removed once they were discovered. In general, World health organization (WHO) susceptibility test procedure was followed as described before [9].

#### Data quality control

Each bioassay was repeated three times. The larval containers were thoroughly cleaned between each use and a fresh batch of larvae was used for each replicate. Critical care was taken while measuring the powder and preparing the concentration before the actual work was done. Whenever the mortality in the control was between 5–20%, mortality was corrected using Abbott’s formula and whenever it was >20%, the test was repeated [14]. Physicochemical parameters (pH, temperature, turbidity, humidity) were measured at site with their corresponding measurement unit (multi-parameter measurements).

#### Data processing and analysis

The relationship between dose and mortality was analyzed using log dosage-probit mortality regression line with 95% confidence interval. Estimates of the LC values were calculated using probit analysis program version 1.5. Chi square test was used to determine the level of significance of the effects of treatments on larvae mortality. The alpha value was set at 0.05 with p < 0.05 was considered statistically significant.

### Results

During the entire experiment of the study, a total of 2400 third instars larvae of *A. arabiensis* were used. This study revealed that unpurified seed products of *P. dodecandra* have a lethal effect on the larval stage of the vector. The powder and crude extract form of the seed products were applied on both the laboratory and field strains of the larvae.

#### Larvicidal effect of *Phytolacca dodecandra* seed powder

As it is presented on Table 1, there was a significant relation between the mortality rate of the larvae and the dose of the seed powder. Hundred percent (75 out of 75) of the laboratory strains of the larvae died at 50 mg/l dose of the powder and the LC_99_ values based on probit analysis was found to be 121.077 mg/l. However, the LC values increased for the field population of *A. arabiensis.* For example, 50 mg/l of the powder brought 96% (72 out of 75) mortality rate and the LC_99_ value was elevated to 195.565 mg/l.

**Table 1:** Larvicidal effect of *Phytolacca dodecandra* seed powder against populations of *Anopheles arabiensis*, Ethiopia, 2016

Nature of the larvae	Dose response			LC values and confidence limits				*p* values
	Conc. (mg/l)	No. tested	No. dead	LC	Mean conc.	95% CI		
						Lower	Upper	
Laboratory strain	5.0	75	15	5	3.096	1.632	5.830	0.05
	10.0	75	23	10	4.330	1.658	7.445	
	20.0	75	42	15	5.430	1.735	8.815	
	30.0	75	55	50	14.136	8.656	19.949	
	40.0	75	66	85	36.802	25.383	76.791	
	50.0	75	75	90	46.152	30.571	113.132	
	+cntrl	75	75	95	64.544	39.622	204.145	
	−cntrl	75	0	99	121.077	62.853	633.312	
Field population	5.0	75	9	5	3.889	1.100	7.509	0.05
	10.0	75	20	10	5.566	1.195	9.686	
	20.0	75	30	15	7.089	1.935	11.566	
	30.0	75	44	50	19.706	12.337	29.473	
	40.0	75	55	85	54.783	35.068	168.437	
	50.0	75	72	90	69.775	42.101	271.327	
	+cntrl	75	75	95	99.851	54.553	556.383	
	−cntrl	75	0	99	195.565	87.067	2179.398	

*conc.* concentration, *+cntrl* positive control, *−cntrl* negative control

#### Larvicidal effect of *Phytolacca dodecandra* seed extract

This study showed that the larval mortality rate increased with increasing plant extract concentration. The lowest and the highest mortality rate were observed at 5 and 50 mg/l of the crude seed extract form of *P. dodecandra*, respectively. Unlike the 50 mg/l of powder application, which results in 100% mortality rate on the laboratory populations of the vector, 50 mg/l of the extract form brought 80% (60 out of 75) of the larval death. The LC_99_ values against the laboratory and field populations were 616.461 and 846.394 mg/l, respectively (Table 2).

**Table 2:** Larvicidal effect of *Phytolacca dodecandra* seed extract against populations of *Anopheles arabiensis*, Ethiopia, 2016

Nature of the larvae	Dose response			LC values and confidence limits				p values
	Conc. (mg/l)	No. tested	No. dead	LC	Mean conc.	95% CI		
						Lower	Upper	
Laboratory strain	5.0	75	9	5	3.694	1.179	8.089	0.05
	10.0	75	15	10	5.900	1.590	11.096	
	20.0	75	21	15	8.092	1.301	13.890	
	30.0	75	31	50	30.764	19.634	67.537	
	40.0	75	40	85	116.962	57.651	168.177	
	50.0	75	60	90	160.422	71.143	377.243	
	+cntrl	75	75	95	256.198	96.495	1255.738	
	−cntrl	75	0	99	616.461	169.017	12,070.555	
Field population	5.0	75	5	5	4.899	2.790	6.981	0.05
	10.0	75	11	10	7.849	5.149	10.315	
	20.0	75	19	15	10.789	7.741	13.504	
	30.0	75	27	50	41.390	34.399	53.176	
	40.0	75	33	85	158.789	106.918	299.344	
	50.0	75	49	90	218.258	138.593	454.521	
	+cntrl	75	75	95	349.671	203.213	845.383	
	−cntrl	75	0	99	846.394	415.301	2715.533	

*conc.* concentration, *+cntrl* positive control, *−cntrl* negative control

The present study also conducted an experiment to assess the lethal effect of *P. dodecandra* seed products on non-target aquatic macro invertebrate organisms (Ceratopogonidae and Coenagrionidae). Dead macro invertebrates were identified by using microscope and family level identification key. The lethal dose of the plant products, which had resulted in 99% of larvae mortality of *A. arabiensis*, had brought only up to 4% mortality rate on the non target organisms.

### Discussion

The extensive usage of synthetic chemicals as mosquito control approach leads to the development of significant resistance to the majority of the chemicals that are available so far [3, 5–7]. Moreover, chemicals are not generally safe to the environment as they have hurtful effects on non target organisms [4]. However, there exists challenges for mosquito control using synthetic chemicals, there are opportunities for extra formulation of tools to manage the menace. Hence, it calls for researchers to invest their knowledge and search for alternative agents that are more efficient, cost-effective, and biodegradable natural insecticides from botanicals.

**Fig. 1:**
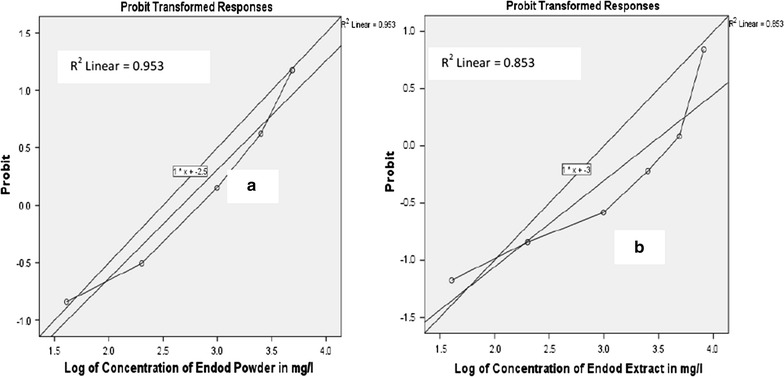
Larvicidal effect of *Phytolacca dodecandra* seed powder (**a**) and extract (**b**) against laboratory strain of *Anopheles arabiansis*

It is evident from the present study that, the exposures of *A. arabiensis* larvae to the seed products of *P. dodecandra* elicit larvae mortality. A substantial larvicidal effect of the seed products (both the powder and extract type) against third instars larvae of *A. arabiensis* was observed. In both forms of the seed product preparations, the larval mortality rate increased significantly with increasing in concentration. For example, 0.853 R^2^ value from Log-probit analysis indicated that the 85.3% variation in larval mortality of laboratory strain of *A. arabiensis* might be explained due to the difference in concentration of the seed extract (Fig. 1).

As it is presented on both tables (Tables 1, 2), the required lethal doses for the field population of *A. arabiensis* was a bit higher than those for the laboratory cultivated larvae. In this context, the laboratory strain of *A. arabiensis* might be more delicate and liable to the seed products than the other group of the vector. This can be explained that field populations may develop adaptation to some hazard factors on the external environment. Nevertheless, it should be noted that the crude seed products of the studied plant have a toxic effect on both the laboratory and field populations of third stage larvae of the vector.

**Fig. 2:**
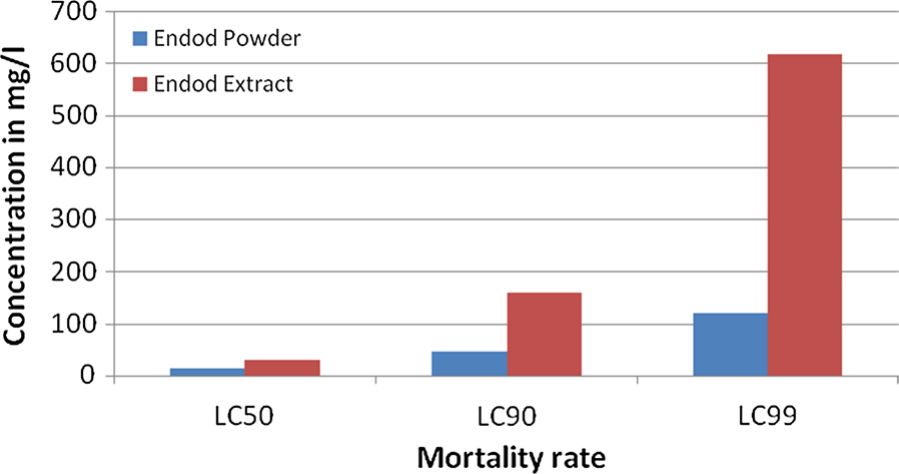
Larvicidal effect of *Phytolacca dodecandra* seed products against laboratory strain of *Anopheles arabiansis*

As far as the lethal potencies of the two preparation forms were concerned, the larvicidal potency of the powder form of the seed product was more powerful than its crude extract form (Fig. 2). This could be related with the application methods of the plant products. For instance, during the use of the crude extract, only drinking and inhalation of the extract might be happened. On the other hand, in the case of powder application, additional direct ingestion of the powder by the active feeding larvae stages of the vector might be occurred and this may in turn increased the dose of the biologically active ingredient within the larvae and this may made the powder form as it had more larvicidal potential than the other preparation. Moreover, the present study exclusively used purified water, which is poor extracting solvent. However, this finding revealed that ‘Endod’ seed products had an excellent larvicidal potency against *A. arabiensis*. Similarly, Getachew D et al. highlighted that, fresh and aged endod berry’s solutions had a significant lethal effect against the 4th-stage larvae of *A. arabiensis* [15]. It is also supported by other previous study that the crude seed products of *P. dodecandra* showed an admirable larvicidal and pupicidal properties against immature *C. quinquefasciatus* [16].

Interestingly, this study also pointed out that the studied plant didn’t have significant lethal effect on non target organisms and this indicated that the plant products may have minimal effects on the non target organisms and works environmental friendly. Similarly, a few scholars had clearly put evidences that; products of *P. dodecandra* had toxic effects on different medically important vectors and parasites and low toxicity to mammals [17, 18].

### Conclusion

The crude preparations of *P. dodecandra* seed products are a promising weapon to control malaria endemic. It can be suitable for temporary mosquito control in small man made breeding places.

## Limitations

The major limitation of the present study is that:

- Its effect was not evaluated on all developmental stages of the vector.
- Besides, due to recourses related constraints, the present study was not able to identify and characterize the bioactive ingredients of its products.

## Abbreviations

LC: lethal concentration
ml: milliliter
mg/l: milligram/liter
SSA: sub-Saharan Africa
WHO: World Health Organization
